# Perinatal Mortality Associated with Positive Postmortem Cultures for Common Oral Flora

**DOI:** 10.1155/2017/9027918

**Published:** 2017-02-23

**Authors:** Mai He, Alison R. Migliori, Patricia Lauro, C. James Sung, Halit Pinar

**Affiliations:** ^1^Department of Pathology & Laboratory Medicine, Women & Infants Hospital of Rhode Island, Providence, RI, USA; ^2^Department of Pathology & Laboratory Medicine, Warren Alpert Medical School of Brown University, Providence, RI, USA; ^3^Department of Pathology and Immunology, Washington University School of Medicine, St Louis, MO, USA

## Abstract

*Introduction*. To investigate whether maternal oral flora might be involved in intrauterine infection and subsequent stillbirth or neonatal death and could therefore be detected in fetal and neonatal postmortem bacterial cultures.* Methods*. This retrospective study of postmortem examinations from 1/1/2000 to 12/31/2010 was searched for bacterial cultures positive for common oral flora from heart blood or lung tissue. Maternal age, gestational age, age at neonatal death, and placental and fetal/neonatal histopathological findings were collected.* Results*. During the study period 1197 postmortem examinations (861 stillbirths and 336 neonatal deaths) were performed in our hospital with gestational ages ranging from 13 to 40+ weeks. Cultures positive for oral flora were identified in 24 autopsies including 20 pure and 8 mixed growths (26/227, 11.5%), found in 16 stillbirths and 8 neonates. Microscopic examinations of these 16 stillbirths revealed 8 with features of infection and inflammation in fetus and placenta. The 7 neonatal deaths within 72 hours after birth grew 6 pure isolates and 1 mixed, and 6 correlated with fetal and placental inflammation.* Conclusions*. Pure isolates of oral flora with histological evidence of inflammation/infection in the placenta and fetus or infant suggest a strong association between maternal periodontal conditions and perinatal death.

## 1. Introduction

Infection is a leading cause of preterm birth, stillbirth, and neonatal death [1–5]. Both infection and the inflammatory response play significant roles in the pathogenetic processes leading to adverse pregnancy outcomes. Most intrauterine infection is caused by bacteria, most commonly species that are normally part of the genital tract flora. Nongenital tract organisms such as those found in the oral cavity can also populate the intrauterine environment via hematogenous spread or oral-genital contact [3, 5]. Therefore, the potential sources of infection can be both the mother and her partner. Possible associations between maternal periodontal diseases and adverse pregnancy outcomes have attracted considerable attention [3, 6–9].

Intrauterine infection and the inflammatory response can be examined via different approaches. These include culture- and nonculture based detection of microorganisms and the measurement of inflammatory cytokines in amniotic fluid, placenta, and blood or tissue from the mother or neonate. In the case of autopsy, histological examination for features of infection and/or inflammation can be performed in placental or in fetal tissue. In a simplified way, chorioamnionitis can be regarded as evidence of a maternal inflammatory response. Funisitis, the inflammation of the umbilical cord, and vasculitis of fetal vessels in the fetoplacental unit can be regarded as histological markers for fetal inflammatory response. The infected amniotic fluid can be swallowed by the fetus in utero, depositing inflammatory cells in the lungs and gastrointestinal tract. The presence of maternal and fetal inflammatory responses, inflammatory cells in fetal tissue, and/or positive fetal tissue and blood cultures are sometimes referred to as amniotic fluid infection syndrome (AFIS) [10].

Previous studies have applied these approaches to examine the relationship between maternal periodontal conditions and amniotic fluid or fetal blood cultures from preterm births and stillbirths [9, 11–13]. Goepfert et al. compared maternal oral conditions with placental histological findings and placental/cord blood cultures but found that neither were associated with periodontal disease [13]. However, while periodontal disease has been shown to be a risk factor for stillbirth [14, 15], there are very few studies looking into the presence of common oral flora in postmortem bacterial cultures in cases of stillbirth or neonatal death within 72 hours of birth [9, 12].

We hypothesize that if oral flora is involved in intrauterine infection and subsequent stillbirth or neonatal death, these bacteria might be present in fetal or neonatal tissue or blood. The current study was aimed at exploring the potential association between oral flora and adverse pregnancy outcomes by investigating bacterial culture results from perinatal autopsies. The study was further correlated with histological features of infection and inflammation of the placenta and fetal/neonatal tissue.

## 2. Materials and Methods

This was a single-institute retrospective study conducted via chart review (IRB approval No. 10-0129). Our hospital is a tertiary care center for pregnant women in which more than 80% of the deliveries statewide take place. Postmortem examinations of stillborns and neonates were performed following the standard division protocol, including the sampling from heart blood cultures and routine lung tissue cultures [16].

### 2.1. Bacterial Culture and Identification

Patient bacterial specimens are cultured on the various media using a standardized method. The lung culture is performed on BAP (Blood agar with 5% sheep blood), MacConkey agar, Chocolate agar, reducible BAP, and Thioglycollate broth. All plates are incubated for 18–24 hours at 35°C in 5% CO_2_, except for reducible BAP, which is incubated in an anaerobe jar at 35°C. All lung culture plates are incubated for 4 days and examined daily.

Blood cultures consist of a pediatric aerobic bottle and an adult anaerobic bottle. If a scant amount of blood is obtained, only the pediatric aerobic bottle will be inoculated, which is effective with as little as 1 mL of blood. The blood culture bottles are incubated in the BacT/Alert® (BioMerieux) continuous monitoring blood culture system for 5 days. Positive blood cultures are transferred to BAP, Chocolate agar, and MacConkey agar plates. These plates are incubated for 18–24 hours at 35°C in 5% CO_2_. A reducible blood agar plate is inoculated and incubated in an anaerobic jar at 35°C.

The Vitek 2® (BioMerieux) automated identification system is used to identify most Gram-negative enteric bacilli,* Pseudomonas,* and other nonlactose fermenting Gram-negative bacilli and Gram-positive cocci such as* Staphylococcus* species and* Streptococcus* species. Yeast isolates are also identified on the Vitek 2 system. The RapID™ System is used for identification of anaerobic bacteria, Gram-positive bacilli,* Haemophilus* species, and* Neisseria* species.

### 2.2. Study Design

After receiving approval from the Institutional Review Board (IRB), records of postmortem examinations (PMs, autopsies) performed during 1/1/2000 to 12/31/2010 were searched for positive bacterial cultures from fetal heart blood or fetal lung tissue. Relevant clinical information including maternal age, gestational age (GA) at birth, chronological age at neonatal death, and placental and fetal/neonatal histopathology was collected for each of these cases.

Common oral flora, as suggested by Socransky et al. [17], includes known periodontal pathogens such as* Aggregatibacter actinomycetemcomitans*,* Porphyromonas gingivalis*,* Tannerella forsythia*,* Treponema denticola*,* Fusobacterium nucleatum*,* Prevotella intermedia*,* Eikenella corrodens*, and* Eubacterium nodatum*; Gram-positive bacteria such as* Streptococcus sanguis*,* mutants, mitis*, and* salivarius*; and other Gram-negative anaerobic bacteria such as* Campylobacter rectus*.

## 3. Results

During the study period, 1197 PMs (861 stillbirths and 336 neonatal deaths) were performed in our hospital with GA ranging from 13 to 40+ weeks. Among these, 227 (19%) yielded positive blood and/or lung cultures, including 165 stillbirths and 62 neonates. Positive cultures for oral flora were identified in 24 cases including 18 pure and 8 mixed growths (more than one species isolated; 26/227, 11.5%), found in 16 stillbirths and 8 neonatal deaths in the following summary of bacterial cultures in postmortem examination of stillbirth and neonatal death from 2000 to 2011.

*Bacterial Cultures in Perinatal Autopsies*. There were 1197 cases of postmortem examinations with gestational age from 13 to 40+ weeks:

- 861 stillbirths (S) and 336 neonatal deaths (N),
- 227 (19%, 165S and 62N) with positive postmortem bacterial cultures,
- 24 (10%, 16S and 8N) cases with positive cultures of oral flora (Some autopsy cases yielded more than one positive bacterial culture),
- 18 cultures growing pure bacterial species,
- 8 cultures growing mixed bacterial species.

*Histopathology-Bacterial Cultures Correlations*. There were 16 stillbirths (median gestational age 22 weeks):

- 8 AFIS,
- 5 with placental inflammation only,
- 3 without histological features of infection or inflammation.

There were also 8 neonatal deaths:

- 7 died within 72 hours of birth,
- 6 AFIS,
- 6 with pure bacterial isolates,
- 1 with mixed culture results.

Among these 24 cases, 19 (79.2%) exhibited histological features of infection and inflammation in fetal and/or placental tissue.

Of the 16 stillbirth autopsies (median GA 22 weeks), 16 postmortem bacterial cultures grew oral flora species. Microscopic examination of fetal and placental tissue in these cases revealed 8 with AFIS, 5 with placental inflammation, and 3 with no histological inflammation. The 7 neonatal deaths within 72 hours after birth (median GA 21 weeks) grew 6 pure isolates and 1 mixed culture, and 6 cases had AFIS. The clinicopathological findings of all 24 cases are summarized in Table 1.

**Figure 1:**
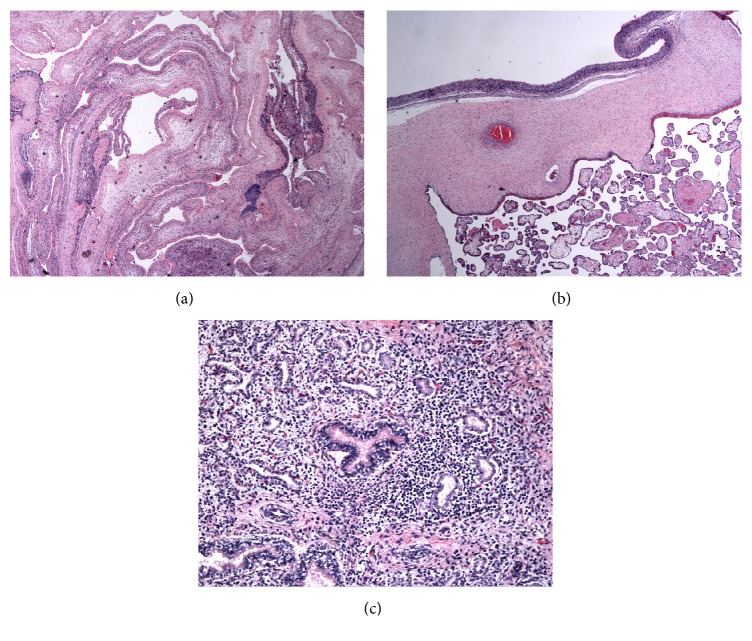
Histopathology of a neonatal death born at 20 weeks with postmortem blood cultures growing* Streptococcus mitis*. (a), (b) Acute necrotizing chorioamnionitis (H&E, 40x). (c) Bronchopneumonia (H&E, 200x).

**Figure 2:**
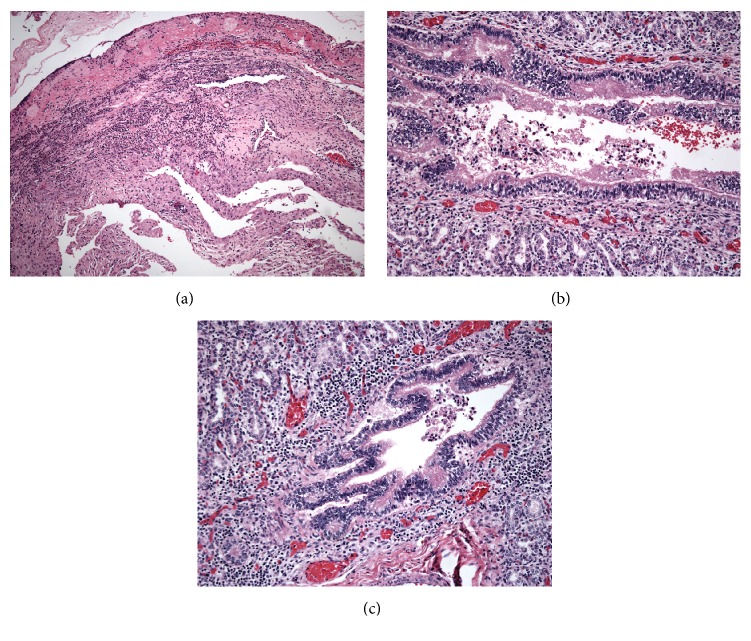
Histopathology of a neonatal death born at 23 weeks to a 14-year-old mother with bleeding gums, postmortem blood, and lung cultures growing* Eikenella corrodens*. (a) Acute necrotizing chorioamnionitis (H&E, 40x). (b), (c) Bronchopneumonia (H&E, 200x).

Figures 1 and 2 demonstrate representative microscopic pictures of both placenta and fetal tissue. Acute inflammation is present in the placental membranes and chorionic plate, consistent with acute chorioamnionitis and suggestive of maternal inflammatory response. When fetal blood vessels at the chorionic plate or umbilical cord are involved, fetal inflammatory response is suggested.

The frequencies of isolated microbes and their microbiological-histological correlations are reported in Table 2.* S. mitis* was isolated from 9 cultures of 6 cases, including 4 stillbirths and 2 neonatal deaths within 4 hours. In 3 cases, there were pure bacterial isolates associated with histological features of infection/inflammation in both fetal and placental tissue (i.e., AFIS).* Peptostreptococcus* species were isolated from 5 cases with one stillbirth growing a pure culture and showing features of AFIS. One stillbirth and one immediate neonatal death at midgestation exhibited* Prevotella*-associated AFIS. Three cultures grew* S. sanguis* including one pure isolate associated with AFIS in a stillbirth. Other microbes were found in single cases.

## 4. Discussion

During the study period of 11 years, bacterial species considered to be common oral flora were identified in 26 cultures from 24 autopsies, yielding an incidence of 2% (24/1197). Eleven autopsies grew pure oral bacterial species and had histological features of infection or inflammation in both fetal and placental tissue, suggesting an association between cultures positive for oral flora and intrauterine infection in these cases of stillbirth or immediate neonatal death.

The most common species isolated was* Streptococcus mitis*, which was isolated from nine cultures in 6 cases (Figure 1 and Table 2).* Streptococcus mitis* is a type of group D* Streptococcus* and falls under the umbrella of* viridans *group* streptococci*. The oral streptococcal group (*mitis* phylogenetic group) currently consists of nine recognized species, although the group has been traditionally difficult to classify, with frequent changes in nomenclature over the years. There are several reports of severe neonatal infection by this group with resultant demise [18]. Interestingly, these neonatal infections occurred within 72 hours after birth, implying that these bacteria may have had a maternal origin.

*S. sanguis* is part of the normal oral flora and alters dental plaque to make it less habitable to other strains of* Streptococcus* that cause tooth decay.* S. salivarius* was isolated from one stillbirth as a pure isolate from both blood and lung tissue. The diagnosis at autopsy was amniotic fluid infection syndrome (AFIS).* S. salivarius* colonizes the mouth and upper respiratory tract shortly after birth and is therefore the principle commensal bacterium of the human mouth. It was isolated as a pure culture associated with AFIS.* S. anginosus *was isolated in a mixed culture from the blood in a case of twin-twin transfusion syndrome. Although it appears that contamination occurred in the current case, this species has previously been seen in autopsy, placental, and fetal tissue bacterial cultures from midgestation abortions [19].* S. mutans* is commonly found in the human mouth and is the primary cause of cavities and tooth decay. In the current study,* S. mutans* was found as a pure isolate from the blood obtained from a 15-week stillborn. The placenta demonstrated features of both maternal and fetal inflammatory response. Although we did not investigate* viridans *group* streptococci *in the current study, a previous study claimed it is a cause of neonatal sepsis, second in frequency only to GBS [20]. There is a case series from our institute in which cultures from 18 perinatal autopsies grew* S. viridans* during a 14-year period [21].

Besides the* Streptococcus *species, the second most common isolates in our study were* Peptostreptococcus *species, which were found in five cases (one pure and four mixed). This is a genus of anaerobic, Gram-positive commensal organisms that colonize the mouth, skin, gastrointestinal tract, vagina, and urinary tract.* Prevotella* species, formerly known as* Bacteroides melaninogenicus*, are human pathogens associated with periodontal disease and upper respiratory tract infections. In the current study,* Prevotella* species were isolated from the fetal lung of an AFIS case, from the lung of a 31-week stillbirth in which no placenta was available for examination, and from a placental culture from a 22-week stillbirth exhibiting maternal and fetal inflammatory response in the placenta.

*Fusobacterium* is a genus of anaerobic, Gram-negative bacilli that is commonly found in the human oropharynx. In our study it was a pure isolate from fetal blood in two cases of AFIS. These species are pathogens that are associated with not only periodontal disease, but also ulcerative colitis and colon cancer. Han et al. identified the same* Fusobacterium* 16s rDNA in supra- and subgingival plaque samples and in the fetal lung and stomach, thus establishing that the source of fetal infection was this microorganism [9]. Heller et al. reported finding filamentous organisms consistent with* Fusobacterium* sp. in the placenta using Warthin-Starry stains in three cases of stillbirths during a 2-year period in one hospital. There was no microbiological identification [22]. In another study, Han considered* F. nucleatum *to be the most prevalent oral species associated with adverse pregnancy outcome [23].

Kostadinov and Pinar, also from our institute, reported a case of neonatal death associated with* Eikenella corrodens *[12].* Eikenella corrodens *is part of the oral flora and often seen in infections involving human bite wounds (Figure 2).

There is increasing evidence to support an interaction between maternal oral flora and the intrauterine environment; pregnancy can lead to an alteration of oral bacterial conditions, and oral flora may affect the outcome of pregnancy [24, 25]. Oral-genital contact further complicates the situation. Previous studies revealed that common oral bacteria have been isolated from amniotic fluid [26] and placenta [27], and there are several case reports of stillbirths whose postmortem cultures grew oral flora [9, 12]. Microbiological-histological correlation analysis is necessary to establish the causal relationship between oral bacterial infection and stillbirth. This study demonstrated that common oral flora species can be isolated from postmortem bacterial cultures in cases of stillbirth or neonatal death. These isolates in addition to histological evidence of inflammation in both placenta and fetal/neonatal tissue suggest a strong association between the presence of oral flora in the intrauterine environment and perinatal death.

Since oral health and dental charts are not included in the practice of prenatal care, one of the limitations of this study was the lack of data on the maternal periodontal conditions. Given the probable association between maternal oral flora and fetal/neonatal demise, it may be advisable to include oral and dental assessments as part of prenatal management.

The most significant limitation to the current study was the reliance on bacterial cultures; thus we were not being able to elucidate the occurrence of fastidious bacteria in our cases (i.e., species that cannot be cultured using standard laboratory methods). Han et al. reported an uncultivated oral* Bergeyella* strain in the amniotic fluid in a case of preterm birth [28]. DiGiulio et al. used a combination of ribosomal DNA identification techniques and conventional cultures to report a greater prevalence and diversity of microbes in amniotic fluid compared to those identified by culture alone. They were able to establish an association between positive PCR results and histological chorioamnionitis and funisitis, as well as a causal relationship between amniotic fluid microbes and preterm labor [29]. Molecular detection of microbes may also be used to reveal similar associations by testing samples from both the oral cavity and amniotic fluid [30, 31]. Histological detection of microorganisms can be used for molecular identification as well [32].

Thus, a future prospective study combining molecular detection techniques and conventional cultures to identify bacteria in the maternal oral cavity, amniotic fluid, and fetal tissue, correlated with histological studies of placenta (and the fetus in cases of stillbirth), could provide more convincing evidence of a causal relationship between intrauterine infection by oral bacteria and stillbirth. This knowledge probably could further contribute to the improvement of pregnancy care.

## Figures and Tables

**Table 1:** Autopsy diagnosis, placental pathology, and bacterial culture results.

Case	Main autopsy diagnosis	Placental finding of inflammation	Blood culture	Lung culture	Other cultures	Mode of delivery	GA	Fetal gender	Length of survival	Mat Age	Maternal medical or obstetric history
1	AFIS, dysmorphic features, and normal CG	Acute chorioamnionitis. Acute vasculitis of chorionic plate.	*S. sanguis*	None	None	Unk	17	F	0	35	Abnormal Thrombophilia. 3 pregnancy losses: first loss at 22 weeks; Turner's syndrome; this is the 2nd loss. 3rd with normal CG.
2	Extreme prematurity, AFIS	Acute chorioamnionitis. Gram-positive cocci seen on Gram stain.	*S. mitis*	No growth	None	VD	20	M	15 min	35	Gestational diabetes mellitus
3	AFIS	Necrotizing acute chorioamnionitis. 3-vessel acute vasculitis and funisitis of the umbilical cord.	*S. salivarius*	*S. salivarius*	None	VD	19	F	0	29	
4	IUFD cause cannot be determined	None	No growth	*Enterococcus species, Peptostreptococcus*	None	Unk	16	F	0	34	Maternal obesity
5	AFIS	Acute chorioamnionitis. 3-vessel acute vasculitis and funisitis of the umbilical cord. Acute vasculitis of chorionic plate.	*S. mitis*	*S. mitis*	None	VD	35	F	0	21	
6	AFIS, extreme prematurity	Acute chorioamnionitis.	*Prevotella species*	No growth	None	VD	22	M	15 min	19	
7	Dysmorphic features, AFIS	Necrotizing acute chorioamnionitis. 3-vessel acute vasculitis and funisitis of the umbilical cord. Acute vasculitis of chorionic plate.	Not taken	*S. sanguis, S. viridans group, P. anaerobius*	None	VD	19	F	0	37	
8	Intrauterine infection	Acute chorioamnionitis. Acute vasculitis and funisitis of the umbilical cord.	*Peptostreptococcus species*	No growth	*Prevotella* from placenta	VD	22	M	0	20	
9	Undetermined	None	*S. mitis*	No growth	None	VD	21	F	0	30	
10	AFIS	Evidence of amniotic fluid infection with fetal inflammatory response.	*Fusobacterium species*	No growth	None	VD	22	M	0	24	
11	Trisomy 18	No placenta submitted	*S. epidermidis*	*S. epidermidis, S. mitis*	None	C/S	36	F	22 days	31	
12	Extreme prematurity, AFIS	Severe necrotizing acute chorioamnionitis. Acute vasculitis and funisitis of the umbilical cord.	*Fusobacterium*	None	None	VD	22	F	5 hours	22	
13	AFIS	Necrotizing acute chorioamnionitis. 3-vessel acute vasculitis and funisitis of umbilical cord. Acute vasculitis of chorionic plate.	Not taken	*1+Staphylococcus coagulase negative. 3+ Peptostreptococcus species and 4+ Lactobacillus species*	None	VD	40+	F	0	20	No prenatal care (unaware of pregnancy). Renal failure and DVT. On weight loss medication.
14	AFIS	Acute chorioamnionitis. 3-vessel acute vasculitis and funisitis of the umbilical cord. Acute vasculitis of chorionic plate.	Anaerobic Gram(−) bacteria, fusiform type, fastidious	No growth	None	VD	38	F	0	30	
15	Twin-twin transfusion syndrome	None	*S. anginosus and Peptostreptococcus*	*Staphylococcus coagulase negative*	None	C/S	30	Unk	0	24	
16	IUGR, abruption, intrauterine infection, and no inflammation seen in fetal tissue	Evidence of intrauterine infection with fetal inflammatory response.	*S. mitis and E. faecalis*	None	None	VD	28	M	0	33	Previous pregnancy loss at 23 weeks of GA. Antiphospholipid syndrome, prophylactic heparin, and aspirin.
17	Intrauterine infection with fetal inflammatory response, no inflammation seen in fetal tissue	Acute chorioamnionitis. 1-vessel acute vasculitis of umbilical cord.	*E. faecalis and S. mitis*	None	GBS in urine	VD	27	F	0	35	Ampicillin for GBS in urine. Prior pregnancy-induced hypertension with full term delivery. Hypothyroidism, on levoxyl daily.
18	Intrauterine infection with fetal inflammatory response, no inflammation seen in fetal tissue	Acute chorioamnionitis. 1-vessel acute vasculitis of umbilical cord. Acute vasculitis of chorionic plate.	*S. mutans*	None	None	VD	15	F	0	23	G3P0. Cervical cerclage. Two previous second trimester losses
19	Extreme prematurity, no inflammation seen in fetal tissue	Acute chorioamnionitis. 1-vessel acute vasculitis and funisitis of umbilical cord. Acute vasculitis of chorionic plate.	*S. mitis*	None	None	VD	19+	M	3 hours 40 minutes	33	
20	AFIS	Acute necrotizing chorioamnionitis of twin A	*E. corrodens*	*E. corrodens*	None	Unk	23	F	>10 minutes	14	Bleeding gums
21	AFIS	Acute chorioamnionitis. 3-vessel acute vasculitis and funisitis of umbilical cord. Acute vasculitis of chorionic plate.	*S. mitis*	*S. mitis*		Unk	36+	M	0	32	
22	AFIS	Acute chorioamnionitis. 2-vessel acute vasculitis and funisitis of umbilical cord.		*S. mitis, S. sanguis*	None	VD	19+	F	2 hours	30	G4P0. Cervical cerclage. Prophylactic antibiotics
23	AFIS	Acute chorioamnionitis.	None	*Prevotella bivia*	None	Unk	21	M	2 hours	15	
24	Fetal hypoxia	No placenta submitted	None	*Prevotella bivia*	None	C/S	31	M	0	20	Pregnancy-induced hypertension, treated with Labetalol

AFIS, amniotic fluid infection syndrome; CG, cytogenetics; C/S, cesarean section; F, female; GA, gestational age; GBS, group B *streptococcus*; IUFD, intrauterine fetal demise; M, male; Mat, maternal; Unk, unknown; VD, vaginal delivery.

**Table 2:** Microbiological and histological correlation in autopsies with positive cultures for oral flora.

Microbe	Number of cases with positive cultures	Pure culture	Mixed culture	Cases with AFIS	Cases with pure cultures and AFIS	Cases with placental finding of inflammation only	Cases with no histological evidence of inflammation	Note
*S. mitis*	9	5	4	4	3	3	1	Case 11 had no placenta submitted
*Peptostreptococcus*	5	1	4	2	1	1	2	
*Prevotella*	4	4	0	2	2	1	0	Case 24 had no placenta submitted
*S. sanguis*	3	1	2	3	1	1	0	
*Fusobacterium*	2	2	0	2	2	0	0	
*S. salivarius*	1	1	0	1	1	0	0	
*S. anginosus*	1	0	1	0	0	0	0	Case 15 had no placenta submitted
*S. mutans*	1	1	0	0	0	1	0	
*E. corrodens*	1	1	0	1	1	0	0	
